# Validation of aqueous two-phase extraction method

**DOI:** 10.1016/j.mex.2021.101421

**Published:** 2021-06-18

**Authors:** Luisaldo Sandate-Flores, José Rodríguez-Rodríguez, Magdalena Rostro-Alanis, Jorge Alejandro Santiago Urbina, Karla Mayolo-Deloisa, Elda M. Melchor-Martínez, Juan Eduardo Sosa-Hernández, Roberto Parra-Saldívar, Hafiz M.N. Iqbal

**Affiliations:** a Tecnologico de Monterrey, School of Engineering and Sciences, Monterrey 64849 Mexico; b Universidad Tecnológica de los Valles Centrales de Oaxaca, Avenida Universidad S/N, San Pablo Huixtepec, Zimatlán de Álvarez, Oaxaca 71270, Mexico

**Keywords:** Aqueous two-phase systems, High-value compounds, Betalains, Betacyanins

## Abstract

Nowadays, consumer interest in food with natural ingredients has increased. This need has led to the research of new sources and green extraction methods. Betalains are compounds responsible for giving color to cacti fruits. The aim is to obtain low-sugar betacyanins extracts from jiotilla *Escontria chiotilla* using aqueous two-phase systems (ATPS) to color food with the extract. The effect of principal parameters of ATPS (Ethyl alcohol- KH_2_PO_4_/K_2_HPO_4_) as tie-line length (TL;40,50 and 70), phase volume ratios (Vr; 1 and 3) on the partitioning of betacyanins, betaxanthins, total sugars, reducing sugars, and antioxidant activity in the extract was evaluated. The yields were determined from the top and bottom phases of the aforementioned parameters. Multivariate analysis of variance (MANOVA, α = 0.05) showed that TLL and Vr were statistically significant (*P* < 0.05). The lowest bottom sugar yield (25.78 ± 3.14%) corresponds to TLL = 40, Vr = 3. Under these conditions, the corresponding value for betacyanins yield is 62.98±4.52%. For the first time, the ATPS was used to extract betacyanins from cactus fruit.

• *Escontria chiotilla*, as a biological source, contained a high percent of betalains
• Aqueous two-phase systems (ATPS) was statistically optimized
• The developed method enriches the valorization of environmentally related plants waste materials

## Graphical Abstract

**Figure:**
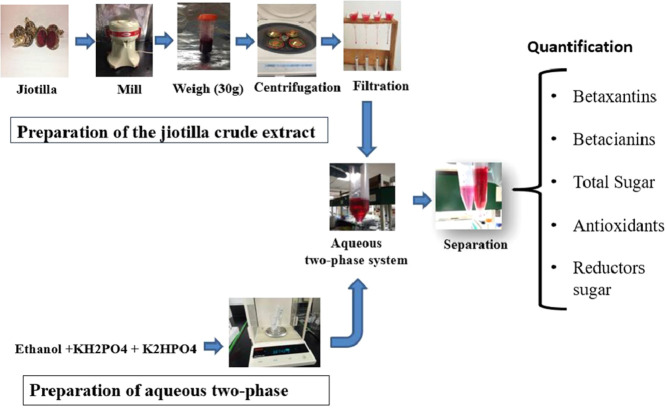

Specifications table

**Table:** 

Subject Area:	Environmental Science
More specific subject area:	*Environmentally related plants waste materials*
Method name:	*Aqueous two-phase systems (ATPS)*
Name and reference of original method:	*NA.*
Resource availability:	*Chemicals/reagents are available from Hycel, Zapopan, Mexico (Item # 1822) and (Item # 40350), Sigma-Aldrich ((Item # P9791), (Item # P3786-500G), and (Item # D9132)), Development of Chemical Specialties (Garcia, Mexico) (Item # DEQA9801000), CTR Scientific (Monterrey, Mexico) (Item # 080.830.5288.1001), and Tedia High Purity Solvents (Fairfield, OH) (Item # MS1922).* *Spectrophotometer (Model DR 500, Hach Lange GmbH, Düsseldorf, Germany).* *Multivariate analysis of variance (MANOVA, α = 0.05) was performed using SPSS (Version 19, IBM Corp, Chicago, IL)*

## Data description – background

Currently, consumer interest in food with natural ingredients has increased [1]. Different natural pigments such as betalains, anthocyanins, and carotenoids are used in the food industry. Betalains are compounds responsible for giving the color to *Beta vulgaris* [2] and cacti fruits such as *Opuntia* [3]*, Hyelocereus* [4]*, Stenocereus* [5]*, and Escontria* [6] genera. Betalains are polyphenolic pigments [7] that are soluble in water [2]. In addition, these compounds are divided into betacyanins (red-violet) and betaxanthins (yellow-oranges) [8]. The main source for betalains at the industrial level is *Beta vulgaris* [9]. However, a disadvantage of beetroot is its earthy flavor [2,10]. The betalains from *Beta vulgaris* could be extracted and purified using high-performance counter-current chromatography (HPCCC). However, this technique has a high investment cost [11]. Because of the high cost, it is necessary to find a viable option with low cost, and one option is the ATPS [12]. This technique does not require high maintenance costs and specialized training [13]. Furthermore, other advantages of ATPS are easy scaling and biocompatibility [13], the technique has been employed in the extraction of pigments. An example of this is obtaining anthocyanins extracted from grape juice by a system NaH_2_PO_4_-ethanol removed sugar [14]. In literature, some works using ATPS have reported removing sugars from betalains in a beetroot extract with a PEG 6000/ammonium sulfate [15]. The betalains were fractioned into betacyanin and betaxanthin in a beetroot extract by PEG 6000-ammonium sulfate system [16]. ATPS was used in pitaya (*Stenocereus pruinosus*) to obtain betaxanthins (PEG-1000-phosphates) [17]. However, the ATPS has never been used to get betanin from the fruit of a cactacea. Betalains from cacti fruit have been used to color food products such as yogurt, gummies, and beverages [18]. There are cacti fruits that are underutilized in Mexico [19], which are sustainable sources to obtain pigments. One of the fruits with the significant potential to obtain these pigments is the “Jiotilla” from *Escontria chiotilla.* Jiotilla plant is a columnar cactus that is endemic of Mexico [20]. The betalains found in jiotilla were vulgaxanthin I, vulgaxanthin II indicaxanthin and betanin [6]. The use of methanol [6] and acidified mucilage [21] have been reported as extraction methods to obtain betalains from Jiotilla*.* Antioxidant compounds have been reported in the fruits of cacti such as *p*-coumaric, caffeic acid [22], and gallic [22], [23], [24], [25], [26], [27], [28]. Antiproliferative properties against prostate and pancreas cancer lines have been found in different plant extracts, including jiotilla [24], [25], [26], [27], [28], [29]. For this reason, it is important to monitor the antioxidant capacity in the extracts. This work looked a green extraction method that was practical and easy to implement in communities. For the extraction process to be applied in the communities, the following conditions must be met: Cheap chemical reagent; the solvent could be easily extracted and recycled; the extraction method does not require specialized training; low investment costs. The ATPS has the aforementioned characteristics in the extraction method, this process could be used in communities to generate incomes. Therefore, the aim of this research is to obtain low-sugar betacyanins extracts from *Escontria chiotilla* using ATPS. Likewise, the effects of the main parameters of ATPS will be evaluated, those parameters are tie line length (TLL), phase volume ratio (Vr) on the partitioning of betacyanins, betaxanthins, total sugars, reducing sugars and antioxidant activity. The extract could be used in the food industry, to color foods such as jellies, gums and ice creams.

## Method validation

### Aqueous two-phase systems (ATPS)

**Fig. 1:**
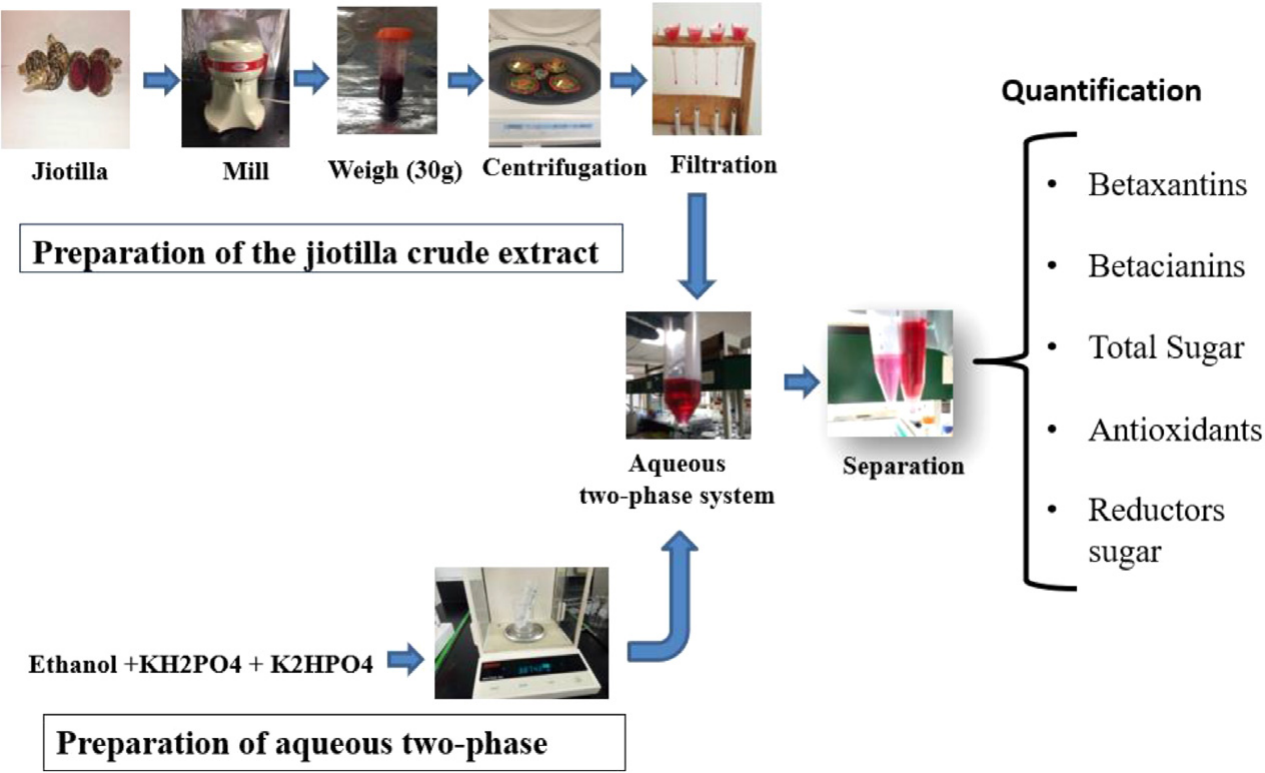
Scheme based on aqueous two-phase system extraction of betacyanins.

ATPS was prepared based on the methods reported by Gomez-Loredo et al. [30]. The conditions tested were TLL = 40, 50 and 70 % w/w, and 10% w/w of crude jiotilla extract (filtrate). Phase volume ratios (Vr) were calculated as the ratio of the volumes of the top (Vt) and bottom (Vb) phases. Vr used were 1 and 3. All experiments were carried out at 25 ± 2 °C. After the crude extract was added, the systems were mixed for 15 min in a tube rotator (50 rpm, Model 05-450-200, Fisher Scientific, Shanghai, China). The top phase was removed using a Pasteur pipet, and the volume and weight of each phase were measured. Fig. 1 shows the extraction scheme used.

### Yields (% w/w) in top and bottom phases

Yields of betacyanins (*BcY_t_* and *BcY_b_*), betaxanthin (*BxY_t_* and *BxY_b_*), total sugars (*SY_t_* and *SY_b_*), antioxidant activity (*AY_t_* and *AY_b_*) and reducing sugars (*RsY_t_* and *RsY_b_*) were determined from the top (subindex *t*) and bottom (subindex *b*) phase content (mg) using Eqs. 1 and 2. Where $TY_{t}$ and $TY_{b}$ can be one of the aforementioned yields. $NTC_{t}$ and $NTC_{b}$ are the content of betacyanins/betaxanthins/total sugars/antioxidant activity/ reducing sugars in the top and bottom respectively [16].

(1) $$TY_{t}=\frac{NTC_{t}}{NTC_{t}+NTC_{b}}\cdot 100$$

(2) $$TY_{b}=\frac{NTC_{b}}{NTC_{t}+NTC_{b}}\cdot 100$$

### Experimental design and statistical analysis

The experimental design included the factors Vr (1, and 3), and TLL (40, 50, and 70% w/w) tested with three replicates. Multivariate analysis of variance (MANOVA, α = 0.05) was performed using SPSS (Version 19, IBM Corp, Chicago, IL).

## Method results and discussion

**Fig. 2:**
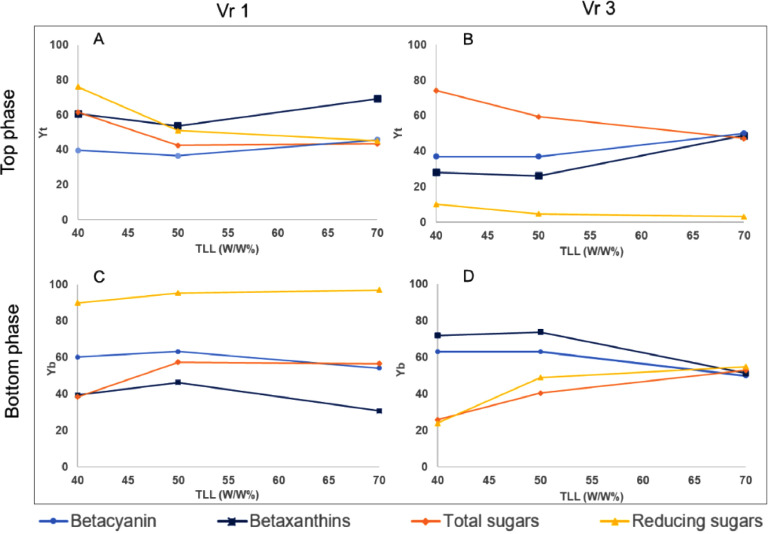
Estimated marginal means of yields (statistical test) in top and bottom phases are represented as follows: betacyanin yield $\left(BcY_{t}\right)$ with sky blue and circle, betaxanthin yield$\left(BxY_{t}\right)$ with blue and squares, total sugars yield$\;(SY_{t}\;$) with orange color and diamond, reducing sugars $\left(RsY_{t}\right)$ yellow and triangles. TLL means tie line length, Yt means yields in top phase, Yb means yields in bottom phase, Vr 1 means volume ratio 1, Vr 3 volume ratio 3. In subsection A, Vr 1 is found in the top phase. In subsection B, Vr 3 is found in the top phase. In subsection C, Vr 1 is found in the bottom phase. In subsection D, Vr 3 is found in the bottom phase.

The yields were calculated in the top and bottom phases to know how the compounds are concentrated. The effects of 40, 50 and 70% w/w TLL values in the top and bottom phases are summarized in Fig. 2. Our experimental results demonstrate that crude jiotilla (*Escontria chiotilla*) low-sugar betacyanins extracts were obtained using ATPS. The system (Ethyl alcohol- KH_2_PO_4_/K_2_HPO_4_), at our knowledge, it has not been reported in the extraction of betalains. A similar approach (ethanol-NaH_2_PO_4_) was used to extract anthocyanins [13], and the highest yields were found in the top phase, in contrast with this work. Other ATPS and betalains sources have been used for the extraction, such as PEG 6000-ammonium sulfate in beetroot (*Beta vulgaris*) [14,15] and PEG 1000-phosphates in yellow pitaya (*Stenocereus pruinosus*) [16]. In the system before mentioned, betacyanins yields were higher in the bottom phase [14], [15], [16]. If a high concentration of betacyanins had been obtained in the top phase, the ethanol could have recovered and reused [11]. Possible reasons that betacyanins move to the bottom phase: The solubility of compounds and phase hydrophobicity effect. The phase hydrophobicity effect is related to the chemical identity of the components of the systems [24]. Water is a better extraction solvent for betalains than ethanol [8,25]. However, when only water is used in liquid-solid extractions, high concentrations of pectins and mucilage are obtained [8]. ATPS has been used to extract polysaccharides with a similar system (ethanol-ammonium sulfate) [26]. Pectins and mucilage can also interfere with betacyanin yields. Betacyanins are more related to the lower phase where water is found. The following yields were compared in the top phase. In subsection A and B, Vr 1 and Vr 3 show that the increase in tie-line length (TLL) produces an increase in marginal means of betacyanin ($BcY_{t}$) and betaxanthins $\left(BxY_{t}\right)$ yields. This phenomenon could be explained because the majority of betacyanins are concentrated in bottom phase. The betaxanthins yields with Vr 3 have the same behavior that betacyanins, the highest concentration is in the bottom phase. Regarding TLL, an increase in TLL produce an increase in betacyanins and betaxanthin yields. This can be explained by the fact that in the ethanol-phosphate system the top phase is more hydrophobic so a lower Vr will have a higher yield. Marginal means of total sugars yield ($\;SY_{b}$) and reducing sugars ($RsY_{t}$) decrease as TLL increases A and B (subsections Fig. 2). In subsection C and D show that the increase in tie line length (TLL) produces an increase in marginal means of total sugars yield ($\;SY_{b}$) and reducing sugars ($RsY_{t}$). Marginal means of betacyanin yield ($BcY_{t}$) and betaxanthin yield ($BxY_{t}$) decrease as TLL increases in bottom phase. The two factors included in this study, TLL and Vr were statistically significant (*p* < 0.05) (Tables 1 and 2). The exception were betacyanins in Vr (*p* = 0.794) antioxidant activity in TLL (*p* = 0.448) (Tables S1 and S2).

**Table 1:** Multivariate analysis of variance, factors phase volume ratio (Vr) and tie-line length (TLL) in bottom phase, multivariate tests.^a^

Effect	Value	F	Hypothesis df	Error df	Sig.
Intercept	.001	3029.670^b^	5.000	8.000	.000
TLL	.002	30.768^b^	10.000	16.000	.000
Vr	.003	567.016^b^	5.000	8.000	.000
TLL * Vr	.084	3.919^b^	10.000	16.000	.008

a Design: Intercept + TLL + Vr + TLL * Vr

b Exact statistic; Statistic used Wilks´Lambda

**Table 2:** Multivariate analysis of variance, factors phase volume ratio (Vr) and tie-line length (TLL) in top phase, multivariate tests.^a^

Effect	Value	F	Hypothesis df	Error df	Sig.
Intercept	.001	2826.038^b^	5.000	8.000	.000
TLL	.001	43.541^b^	10.000	16.000	.000
Vr	.002	840.903^b^	5.000	8.000	.000
Wilks' Lambda	.002	840.903^b^	5.000	8.000	.000
TLL * Vr	.043	6.090^b^	10.000	16.000	.001

a Design: Intercept + TLL + Vr + TLL * Vr

b Exact statistic; Statistic used Wilks´Lambda

Yields of reducing sugars in the top phase decrease as the TLL increases. No literature was found about ATPS and reducing sugars, but the objective was to eliminate the sugar because the free sugar sugars accelerate the degradation of betalains [14]. The effect of TLL on the yields of antioxidant activity was not statistically significant, but Vr is significant (Table 3). A reason to explain this behavior is that the DPPH antioxidant activity technique measures betalains and other compounds such as phenolic compounds. This could be the reason that antioxidant activity is not statically significant. ATPS could enrich the phenolic compounds in the top phase, and this behavior was observed in the extraction and enrichment of genistein and apigenin using a system 28% ethanol and 22 %K_2_HPO_4_ [27]. However, an increase in antioxidant activity was observed in ATPS (ethanol-ammonium sulfate) with different concentrations of extracts used to obtain anthocyanins from mulberry (*Morus atropurpurea Roxb.)* [28].

**Table 3:** Yields of antioxidant activity.

TLL	Vr	Bottom(%)	Top(%)
40	1	29.31 ± 2.94^b^	70.69 ± 5.22^a^
40	3	66.69 ± 4.51^a^	33.31 ± 2.35^b^
50	1	29.37 ± 3.58^b^	70.63 ± 5.14^a^
50	3	72.55 ± 4.98^a^	27.45 ± 3.82^b^
70	1	26.96 ± 1.72^b^	73.04 ± 5.02^a^
70	3	67.69 ± 8.49^a^	32.31 ± 2.33^b^

Values represented as mean ± standard deviation (*n* = 3); different lowercase letters (a-b) indicate statistical significance difference (*p* < 0.05); the yields of the phases were compared separately; TLL is tie-line length; Vr is volume ratio.

## Conclusion

The aim of this work was achieved, which was to obtain low-sugar betacyanins extracts from *Escontria chiotilla* using ATPS. However, the research was expected to have a higher concentration of betacyanins in the top phase where the ethanol is located to remove the solvent easily. In contrast, the yields of betaxanthins were higher in the top phase than yields of betacyanins. For this reason, the ethanol-phosphate system could be employed in future works for the separation of betacyanins and betaxanthins. The advantages of this system are to obtain two pigments of different colors from the same resource or in fruits with a high concentration of betaxanthins to get the pigment on the top phase. This work showed ATPS could be an excellent method to obtain betalains from cacti fruits.

## Appendix Supplementary materials

Image, application 1
