# Supplementary material for: Validation of aqueous two-phase extraction method
Source: MethodsX. 2021 Jun 18;8:101421. doi: 10.1016/j.mex.2021.101421 (PMC8374489; doi:10.1016/j.mex.2021.101421)
Supplement: Supplementary file 1 [file mmc1.docx]

**Validation of aqueous two-phase extraction method**

Luisaldo Sandate-Flores ^a^, José Rodríguez-Rodríguez ^a^, Magdalena Rostro-Alanis ^a^, Jorge Alejandro Santiago Urbina ^b^, Karla Mayolo-Deloisa ^a^, Elda M. Melchor-Martínez ^a^, Juan Eduardo Sosa- Hernández ^a^, Roberto Parra-Saldívar ^a^*, and Hafiz M.N. Iqbal ^a^*

^a^Tecnologico de Monterrey, School of Engineering and Sciences, Monterrey, 64849 Mexico.

^b^Universidad Tecnológica de los Valles Centrales de Oaxaca, Avenida Universidad S/N, San Pablo Huixtepec, Zimatlán de Álvarez, Oaxaca, 71270, Mexico.

* Corresponding Author’s email address: r.parra@tec.mx (R. Parra-Saldívar); hafiz.iqbal@tec.mx (H. M.N. Iqbal)

**Method details**

***Preparation of the jiotilla crude extract***

Jiotilla fruits (22 kg) was collected from a single crop field in Santa María Zoquitlán, Tlacolula, Oaxaca, Mexico (16°33' latitude North and 96°23' longitude West). This area was selected because jiotilla is produced and thus increase the income in this community in the future. Fruits were processed within 24 h after harvest. Jiotillas were washed, disinfected, peel was removed, and finally pulp seeds were removed using a juice extractor (Model TU05, Turmix MR, Estado de Mexico, Mexico). 11 kg of seedless pulp samples (30 g) were stored at -18 ° C in 50 ml polypropylene centrifuge tubes (Corning®, Tewksbury, MA) and used within 30 days. After thawing in the refrigerator (5 ± 2 °C), the pulp was clarified by centrifugation (3600 g, 4 °C, 20 min, Model SL 40R, Thermo Fisher Scientific, Langenselbold, Germany). The supernatant was filtered through 150 mm Whatman paper grade 4 (Item 1009150, GE Healthcare Life Sciences, Little Chalfont, UK). The filter was washed using 3 mL distilled water [1]. This amount of distilled water was used to avoid diluting the sample. Fruit processing was carried out in the dark to avoid betacyanins and betaxanthins degradation.

***Betacyanins and betaxanthins quantification***

The betacyanins (Bc) and betaxanthins concentrations (Bx) (mg/L) were quantified spectrophotometrically (Model DR 500, Hach Lange GmbH, Düsseldorf, Germany) using Eq.1. The method used for betacyanins and betaxanthins concentration was Sandate-Flores et al. [2]. However, a modification was carried out, distilled water was used instead of acidified water. Where *E_1%_* is the corresponding extinction coefficient for betacyanins (λ=540 nm, *E_1%_* =60000 L mol^-1^ cm^-1^) and betaxanthins (λ=480, *E_1%_* =48000 L mol^-1^ cm^-1^) [3], *A* is the corresponding absorbance at 480 and 540 nm, *M* is the molecular weight of representative compounds, f*_d_*  is the dilution factor, *l* is the cuvette pathlength (1 cm). Eq. 2 was used for betacyanins (Bc) and betaxanthins content (Bx) (mg), where *V* is the volume of the extract in the bottom or top phase.

|  | $B=\frac{Axf_{d}xMx1000}{E_{\text{1\%}}l}$ | *(1)* |
| --- | --- | --- |
|  | $Bn=B\times V$ | *(2)* |

***Total sugars quantification***

Dubois et al. method was used to determine total sugar content [4] [31]. Sample aliquots (400 µL) diluted 75-fold were added to 1.5 mL Safe-Lock^TM^ tubes (Eppendorf AG, Hamburg, Germany) and mixed with sulfuric acid (1 mL) and 8% w/w phenol solution (100 µL). The solution was agitated for 10 s, allowed to stand for 10 min, then agitated for 10 s, and finally allowed to stand for another 20 min. The absorbance was measured at 490 nm. A standard xylose was used for sugar quantification (Eq. 3).

|  | | $S =\frac{As-I_{c}}{S_{c}}$ | *(3)* | |
| --- | --- | --- | --- | --- |
|  | $TS=\frac{Sc\times f_{d}\times v}{1000}$ | | | *(4)* |

Where *S* is the sample total sugar concentration (mg/L), *A_s_* is the absorbance at 490 nm, and *I*_c_ (= 0.1306) and *S*_c_ (=0.0058) are the intercept and slope of the xylose calibration curve (*R^2^* = 0,988), respectively. The total sugar content (*TS*, mg) in the top and bottom layer was calculated using Eq. 4. where fd is the dilution factor, and vt and vb are the volume and Sct and Scb the total sugar concentration (mg/L) in the top and bottom phase, respectively.

***Antioxidant activity DPPH***

Based on the method described by Brand-Williams et al. to determine antioxidant activity [5] [32]. 0.0148 mg of the DPPH reagent was weighed and, placed in a volumetric flask (25 mL)). Then the volumetric flask was filled with methanol (mother solution). 2 ml of the mother solution were placed in a volumetric flask (50 mL), then the volumetric flask was filled to the mark with methanol (stock solution). The absorbance was measured at 515 nm in a spectrophotometer (Model DR 500, Hach Lange GmbH, Düsseldorf, Germany). The readings were taken in 2.5 mL cuvette, mixing sample aliquots (75 μL) and 2 ml of the DPPH 6.0X10-5 M diluted in methanol. The reaction takes place16 min after mixing reactants. A calibration curve was made using Trolox standard (5-200 ppm) for each of the phases. All measurements were made in triplicate.

***Reducing sugars***

The determination of reducing sugars was carried out by the method dinitrosaIicyIic (DNS) acid reagent [6] [33]. Which is based on the reduction of 3,5 dinitrosalicylic acid to 3-amino-5 nitrosalicylic acid by the free carbonyl group of the reducing sugars [6] [33]. The complex formed was measured at 540 nm; the optical density is proportional to the concentration of sugar reducers. 200 μL aliquots (top phase diluted 10-fold; bottom phase no dilution was made) plus 200 μL of DNS was added. Then samples were heated to boiling, allowing them to react for 5 minutes and immediately afterwards they were transferred to an ice bath, there they were add 400 μL of cold distilled water and stirred. Finally, the samples were read at a wavelength of 540 nm in a spectrophotometer. A blank was prepared by replacing the sample with distilled water. To determine the concentration of reducing sugars through the absorbance data of the samples, a standard curve was prepared using xylose solutions in a concentration range of 0.0 to 0.1 g/L.

|  | | | | | | |
| --- | --- | --- | --- | --- | --- | --- |
| \| **Table S1.** **Tests of between-subjects effects (MANOVA), factors phase volume ratio (Vr), and tie line length (TLL)** **in bottom phase.** \| \| \| \| \| \| \| \| --- \| --- \| --- \| --- \| --- \| --- \| --- \| \| **Source** \| **Dependent Variable** \| **Type III Sum of Squares** \| **df** \| **Mean Square** \| **F** \| **Sig.** \| \| Corrected Model \| S \| 2349.238^a^ \| 5 \| 469.848 \| 17.988 \| .000 \| \| Btx \| 4544.335^b^ \| 5 \| 908.867 \| 58.224 \| .000 \| \| Btc \| 477.121^c^ \| 5 \| 95.424 \| 4.143 \| .020 \| \| RS \| 13607.444^d^ \| 5 \| 2721.489 \| 262.685 \| .000 \| \| Ac \| 7425.305^e^ \| 5 \| 1485.061 \| 41.899 \| .000 \| \| Intercept \| S \| 36905.917 \| 1 \| 36905.917 \| 1412.908 \| .000 \| \| Btx \| 49055.472 \| 1 \| 49055.472 \| 3142.579 \| .000 \| \| Btc \| 62461.095 \| 1 \| 62461.095 \| 2711.782 \| .000 \| \| RS \| 84007.639 \| 1 \| 84007.639 \| 8108.628 \| .000 \| \| Ac \| 42796.652 \| 1 \| 42796.652 \| 1207.445 \| .000 \| \| TLL \| S \| 1653.091 \| 2 \| 826.546 \| 31.644 \| .000 \| \| Btx \| 1199.364 \| 2 \| 599.682 \| 38.417 \| .000 \| \| Btc \| 437.548 \| 2 \| 218.774 \| 9.498 \| .003 \| \| RS \| 1204.622 \| 2 \| 602.311 \| 58.137 \| .000 \| \| Ac \| 44.905 \| 2 \| 22.452 \| .633 \| .548 \| \| Vr \| S \| 561.013 \| 1 \| 561.013 \| 21.478 \| .001 \| \| Btx \| 3234.761 \| 1 \| 3234.761 \| 207.224 \| .000 \| \| Btc \| 1.850 \| 1 \| 1.850 \| .080 \| .782 \| \| RS \| 11920.195 \| 1 \| 11920.195 \| 1150.567 \| .000 \| \| Ac \| 7354.823 \| 1 \| 7354.823 \| 207.506 \| .000 \| \| TLL * Vr \| S \| 135.133 \| 2 \| 67.566 \| 2.587 \| .116 \| \| Btx \| 110.210 \| 2 \| 55.105 \| 3.530 \| .062 \| \| Btc \| 37.724 \| 2 \| 18.862 \| .819 \| .464 \| \| RS \| 482.628 \| 2 \| 241.314 \| 23.292 \| .000 \| \| Ac \| 25.576 \| 2 \| 12.788 \| .361 \| .704 \| \| Error \| S \| 313.447 \| 12 \| 26.121 \|  \|  \| \| Btx \| 187.319 \| 12 \| 15.610 \|  \|  \| \| Btc \| 276.399 \| 12 \| 23.033 \|  \|  \| \| RS \| 124.323 \| 12 \| 10.360 \|  \|  \| \| Ac \| 425.328 \| 12 \| 35.444 \|  \|  \| \| Total \| S \| 39568.601 \| 18 \|  \|  \|  \| \| Btx \| 53787.126 \| 18 \|  \|  \|  \| \| Btc \| 63214.614 \| 18 \|  \|  \|  \| \| RS \| 97739.406 \| 18 \|  \|  \|  \| \| Ac \| 50647.284 \| 18 \|  \|  \|  \| \| Corrected Total \| S \| 2662.684 \| 17 \|  \|  \|  \| \| Btx \| 4731.654 \| 17 \|  \|  \|  \| \| Btc \| 753.520 \| 17 \|  \|  \|  \| \| RS \| 13731.767 \| 17 \|  \|  \|  \| \| Ac \| 7850.632 \| 17 \|  \|  \|  \| \| a. R Squared = .882 (Adjusted R Squared = .833); b. R Squared = .960 (Adjusted R Squared = .944); c. R Squared = .633 (Adjusted R Squared = .480); d. R Squared = .991 (Adjusted R Squared = .987); e. R Squared = .946 (Adjusted R Squared = .923); S is yield of total sugars; Btx is yield of betaxanthins; Btc is yield of betacyanins; RS is yield of reducing sugars; Ac is yield of antioxidant activity. \| \| \| \| \| \| \|   **Table S2. Tests of between-subjects effects (MANOVA), factors phase volume ratio (Vr), and tie line length (TLL) in top phase.** | | | | | | |
| **Source** | **Dependent Variable** | **Type III Sum of Squares** | **df** | **Mean Square** | **F** | **Sig.** |
| Corrected Model | S | 2349.239^a^ | 5 | 469.848 | 10.071 | .001 |
|  | Btx | 4544.320^b^ | 5 | 908.864 | 293.360 | .000 |
|  | Btc | 477.060^c^ | 5 | 95.412 | 3.690 | .030 |
|  | RS | 13608.330^d^ | 5 | 2721.666 | 525.678 | .000 |
|  | Ac | 7425.975^e^ | 5 | 1485.195 | 56.870 | .000 |
| Intercept | S | 53898.106 | 1 | 53898.106 | 1155.286 | .000 |
|  | Btx | 41118.516 | 1 | 41118.516 | 13272.077 | .000 |
|  | Btc | 30395.095 | 1 | 30395.095 | 1175.396 | .000 |
|  | RS | 18070.272 | 1 | 18070.272 | 3490.195 | .000 |
|  | Ac | 47256.602 | 1 | 47256.602 | 1809.524 | .000 |
| TLL | S | 1653.050 | 2 | 826.525 | 17.716 | .000 |
|  | Btx | 1199.433 | 2 | 599.716 | 193.574 | .000 |
|  | Btc | 437.517 | 2 | 218.759 | 8.460 | .005 |
|  | RS | 1204.849 | 2 | 602.425 | 116.356 | .000 |
|  | Ac | 44.861 | 2 | 22.430 | .859 | .448 |
| Vr | S | 561.013 | 1 | 561.013 | 12.025 | .005 |
|  | Btx | 3234.492 | 1 | 3234.492 | 1044.017 | .000 |
|  | Btc | 1.850 | 1 | 1.850 | .072 | .794 |
|  | RS | 11920.709 | 1 | 11920.709 | 2302.434 | .000 |
|  | Ac | 7355.632 | 1 | 7355.632 | 281.658 | .000 |
| TLL * Vr | S | 135.175 | 2 | 67.588 | 1.449 | .273 |
|  | Btx | 110.394 | 2 | 55.197 | 17.816 | .000 |
|  | Btc | 37.693 | 2 | 18.846 | .729 | .503 |
|  | RS | 482.772 | 2 | 241.386 | 46.623 | .000 |
|  | Ac | 25.482 | 2 | 12.741 | .488 | .626 |
| Error | S | 559.841 | 12 | 46.653 |  |  |
|  | Btx | 37.177 | 12 | 3.098 |  |  |
|  | Btc | 310.314 | 12 | 25.859 |  |  |
|  | RS | 62.129 | 12 | 5.177 |  |  |
|  | Ac | 313.386 | 12 | 26.115 |  |  |
| Total | S | 56807.186 | 18 |  |  |  |
|  | Btx | 45700.014 | 18 |  |  |  |
|  | Btc | 31182.468 | 18 |  |  |  |
|  | RS | 31740.732 | 18 |  |  |  |
|  | Ac | 54995.963 | 18 |  |  |  |
| Corrected Total | S | 2909.080 | 17 |  |  |  |
|  | Btx | 4581.497 | 17 |  |  |  |
|  | Btc | 787.373 | 17 |  |  |  |
|  | RS | 13670.460 | 17 |  |  |  |
|  | Ac | 7739.360 | 17 |  |  |  |
|  | | | | | | |
| a. R Squared = .808 (Adjusted R Squared = .727); b. R Squared = .992 (Adjusted R Squared = .989); c. R Squared = .606 (Adjusted R Squared = .442); d. R Squared = .995 (Adjusted R Squared = .994); e. R Squared = .960 (Adjusted R Squared = .943); S is yield of total sugars; Btx is yield of betaxanthins; Btc is yield of betacyanins; RS is yield of reducing sugars; Ac is yield of antioxidant activity. | | | | | | |

References

1. Sandate-Flores, L.; Rodríguez-Rodríguez, J.; Velázquez, G.; Mayolo-Deloisa, K.; Rito-Palomares, M.; Antonio Torres, J.; Parra-Saldívar, R. Low-sugar content betaxanthins extracts from yellow pitaya (Stenocereus pruinosus). *Food Bioprod. Process.* **2020**, 183135, doi:10.1016/j.bbamem.2019.183135.
2. Sandate-Flores, L.; Rodríguez-Rodríguez, J.; Calvo-Segura, S.; Mayorga-Martínez, A.; Parra-Saldivar, R.; Chuck-Hernández, C. Evaluation of different methods for betanin quantification in pitaya (*Stenocereus* spp.). *Agro Food Ind. Hi Tech* **2016**, *27*, 20–25.
3. Nilsson, T. Studies into the pigments in beetroot. *Lantbrukshögskolans Ann.* **1970**, *36*, 179–219.
4. Dubois, M.; Gilles, K.A.; Hamilton, K.; Rebers, P.A.; Smith, F. Colorometric method for determination of sugars and related substances. *Anal. Chem.* **1956**, *28*, 350–356.
5. Brand-Williams, W.; Cuvelier, M.E.; Berset, C. Use of a free radical method to evaluate antioxidant activity. *LWT - Food Sci. Technol.* **1995**, *28*, 25–30, doi:10.1016/S0023-6438(95)80008-5.
6. Miller, G.L. Use of dinitrosaIicyIic acid reagent for determination of reducing sugar. *Anal. Chem.* **1959**, *3*, 426–428.
